# Unexpected Findings in Hereditary Breast and Ovarian Cancer Syndrome: Low-Level Constitutional Mosaicism in *BRCA2*

**DOI:** 10.3390/genes14020502

**Published:** 2023-02-15

**Authors:** Irene Hidalgo Mayoral, Ainhoa Almeida Santiago, Jose Manuel Sánchez-Zapardiel, Beatriz Hidalgo Calero, Miguel de la Hoya, Alicia Gómez-Sanz, Montserrat de Miguel Reyes, Luis Robles

**Affiliations:** 1 Hereditary Cancer Laboratory, Hospital Universitario 12 de Octubre, 28041 Madrid, Spain; 2 Instituto de Investigación Sanitaria Hospital 12 de Octubre (imas12), 28041 Madrid, Spain; 3 Molecular Oncology Laboratory, Instituto de Investigacion Sanitaria San Carlos, Hospital Universitario Clínico San Carlos, 28040 Madrid, Spain; 4 Medical Oncology Service, Hospital Universitario 12 de Octubre, 28041 Madrid, Spain

**Keywords:** HBOC, constitutional mosaicism, *BRCA2*

## Abstract

Hereditary breast and ovarian cancer syndrome (HBOC) is a clinical entity characterized by an increased risk of developing breast and ovarian cancer. The genetic diagnosis is based on the identification of heterozygous germinal variants in HBOC susceptibility genes. However, it has recently been described that constitutional mosaic variants can contribute to the aetiology of HBOC. In constitutional mosaicism, individuals have at least two genotypically distinct populations of cells that arise from an early post-zygote event. The mutational event occurs early enough in development to affect several tissues. It is detected in germinal genetic studies as low variant allele frequency (VAF) variants (<30%) that are generally overlooked during the prioritization process. Constitutional mosaic variants can affect both somatic and germinal cells, and thus can be passed to the offspring and have important consequences for genetic counselling. In this work, we report the c.9648+1G>A mosaic variant in the *BRCA2* gene and propose a diagnostic algorithm to deal with potential mosaic findings identified by Next Generation Sequencing (NGS).

## 1. Introduction

Hereditary breast and ovarian cancer syndrome (HBOC) is a clinical entity characterized by an increased risk of developing breast and/or ovarian tumors at an early age. It is estimated that 5–10% of breast cancers and approximately 25% of ovarian cancers are due to inherited germline mutations in HBOC-related genes that include *BRCA1*, *BRCA2*, *PALB2*, *PTEN*, *RAD51C*, *RAD51D*, *ATM*, *CHEK2*, *BARD1*, *BRIP1*, *CDH1*, *NBN* or *TP53* among others. Together, *BRCA1* (OMIM#604370) and *BRCA2* (OMIM#612555) are responsible for approximately 25% of HBOC cases. Inherited deleterious variants in *BRCA1* and *BRCA2* genes are highly penetrant for HBOC, with lifetime risks (dependent upon the gene and population) up to 57% for breast cancer and up to 40% and 18% for ovarian cancer for *BRCA1* and *BRCA2* carriers, respectively [1]. They are mainly heterozygous variants that are inherited in an autosomal dominant pattern. However, it has recently been described that low-level constitutional mosaicism in *BRCA1* and *BRCA2* can contribute to the aetiology of early-onset HBOC susceptibility [2,3].

The term mosaicism denotes an individual who has at least two populations of cells with distinct genotypes that are derived from a single zygote. Mosaicism results from a mutational event whose timing and the cell lineage affected determine the tissue and cell type distribution of mosaicism [4]. If it occurs at a very early post-zygotic stage, several cell lines are affected, and mosaicism become an integral part of the individual (constitutional mosaicism). Mutated cell lines can derive from any of the three primary germ layers (ectoderm, mesoderm and endoderm [5]), and it may also affect the germline cells due to the possibility the gonadal cell line was derived from mutated embryonic cells. Thus, it may have implications for reproductive risk [6].

Constitutional mosaicism in cancer related genes has been previously described [7], although it appears to be less frequent in some predisposition syndromes such HBOC. In 2013, Delon et al. reported the first case of low-level constitutional mosaicism in BRCA [8]. Since then, only six additional cases have been reported [2,3,9,10,11].

This may be related to the fact that the detection of mosaicism remains challenging due to methodological and interpretative limitations. Thus, genetic laboratories must develop accurate workflows in the laboratory that prevent mosaic variants to be overlooked. Failure to detect mosaicism may result in inappropriate cancer risk assessment, inappropriate clinical management and a substantial difference in recurrence risk assessment.

In this work we report the c.9648+1G>A mosaic variant in the *BRCA2* gene (NM_000059.3), which to the best of our knowledge constitutes the third case of *BRCA2* constitutional mosaicism described in the literature [3,11]. We highlight the role of mosaic *BRCA1* and *BRCA2* variants in HBOC development and highlight the importance of defining protocols that prevent mosaic variants to be missed and to properly characterize them. Diagnostic algorithms must allow ruling out false positive results, establishing the constitutional nature of the variant and defining its implication in tumor development.

## 2. Materials and Methods

### 2.1. Case Report

The proband is a 39-year-old woman who developed at the age of 25 a high-grade intraductal carcinoma of the left breast. Molecular phenotype: Ki-67 30%, immunohistochemistry ER (3+), PR (2+), HER2neu negative (+1). Left mastectomy was performed in 2009 followed by adjuvant Tamoxifen for 5 years. The patient was free of relapse 14 years after diagnosis. Family’s pedigree is not shown since the proband is an adopted child.

In 2019, she was referred to the Familial and Hereditary Cancer Unit of Hospital Universitario 12 Octubre because she met criteria for referral to genetic counselling due to her risk of HBOC according to the Spanish Society of Medical Oncology (SEOM) clinical guidelines. Selection criteria for germline testing include breast cancer <40 years even regardless of family history. Genetic analysis was not informative and included the *BRCA1* and *BRCA2* study by NGS and MLPA. In 2022, she returned to the clinical genetics’ office for multigene panel testing proposal.

### 2.2. Collection of Samples and Clinical Information

Consent for the use of patient’s data, materials and/or test results for research purposes was included in the written informed consent, which was obtained prior to sample collection. The Ethics Committee of the University Hospital 12 Octubre, in accordance with the Declaration of Helsinki, approved the present study.

### 2.3. Molecular Genetics Studies

Germinal testing was performed in a peripheral blood sample. Additional samples were needed to characterize low VAF finding from NGS analysis: buccal swab, as a derivative sample from the endoderm layer; urine, as a derivative sample from the ectoderm layer; and breast tumor sample to validate the role of the variant in tumor development.

DNA extraction from the peripheral blood leukocytes (PBL) was carried out by automated methods using the Maxwell RSC Blood DNA Kit (Promega Corporation, Madison, Wisconsin, USA) technology whereas DNA extraction from oral swab and urine was carried out using the Maxwell RSC Tissue DNA Kit (Promega). Quality of the DNA extracted was determine by spectrophotometry using Nanodrop (Thermo Fisher Scientific, Waltham, MA, USA). A280/260 and A280/230 ratio were checked as indicators of protein and salt contamination.

Targeted genes (covering the entire coding regions and exon-flanking sequences) were captured with a commercial Hereditary Cancer Solution (HCS) kit (SOPHiA GENETICS, Saint-Sulpice, Switzerland). Libraries were quantified by a fluorimetric technique using Qubit dsDNA High Sensitivity kit (Thermo Fisher Scientific) and quality was assessed using the 4150 TapeStation System (Agilent, Santa Clara, California, USA). Paired-end sequencing was carried out on a NextSeq 550 sequencer (Illumina, San Diego, California, USA). Bioinformatic analysis was performed using the SOPHiA-DMM platform that uses a private pipeline based on three tools: Pepper (for SNV and Indel detection), MUSKAT (for CNV identification) and MOKA (variant annotation). NGS data was analysed using a custom panel that is comprised of 18 genes: *ATM*, *BARD1*, *BRCA1*, *BRCA2*, *BRIP1*, *CDH1*, *CHEK2*, *MLH1*, *MSH2*, *MSH6*, *EPCAM*, *NBN*, *PALB2*, *PTEN*, *RAD51C*, *RAD51D*, *STK11*, *TP53*. Genes were selected based on the list of potential genes compiled by Taylor et al. [12] that increase the risk of developing breast and/or ovarian cancers. Variants that are located in low complexity regions or outside the target region, have a variant fraction lower than expected (germline) or have a coverage less than 30X are filtered as low-confidence variants. Variant prioritization was performed using the following criteria: (i) Minor Allele Frequency (MAF) < 1% in gnomAD population database (v2.1.1); (ii) synonymous variants outside the canonical splicing site not described in literature or not supported to disrupt normal splicing by splicing predictors were discarded; (iii) intronic variants localized 15 nucleotides or more from the exon/intron junction not described in literature or not supported to disrupt normal splicing by splicing predictors were discarded; (iv) status and ranking of the variants in the ClinVar database; (v) variant pathogenicity predictors including Sorting Intolerant from Tolerant (SIFT) [13], Polyphen-2 [14] and Mutation Taster [15]; and (vi) phylogenetic conservation assessment using the Phylogenetic *p*-values program (phyloP) [16]. Data related to the variant prioritized has been submitted to ClinVar under the accession number SCV002760187 (https://www.ncbi.nlm.nih.gov/clinvar/variation/VCV000487418.2, accessed on 12 December 2022).

DNA from buccal swab and urine samples were analysed by high-depth NGS in Health in Code (A Coruña, Spain). Targeted region was amplified by PCR amplification and captured by Nextera XT kit (Illumina). Paired-end sequencing was carried out in a MiSeq sequencer (Illumina). Bioinformatic analysis was performed by Health in Code using the Health in Code datagenomics platform.

DNA extraction from breast tumour sample was carried out with the GeneRead DNA FFPE Kit (QIAGEN, Düsseldorf, Germany) following manufacturer’s protocol. *BRCA2* exon 26 donor site was targeted with a commercial solution BRCA Plus FFPE OncoKitDx (Health in Code) that captures the full-coding sequence and exon-intron boundaries of *BRCA1* and *BRCA2*. Paired-end sequencing was carried out in a MiSeq sequencer (Illumina). Bioinformatic analysis was performed by Health in Code data genomics platform.

### 2.4. Confirmation Analysis

*BRCA2* variant was validated by Sanger sequencing. Although mosaic variants may be undetectable with Sanger sequencing, we chose this validation method because the estimated VAF was above its limit of detection. DNA was amplified by polymerase chain reaction (PCR) in the 7500 Fast Real-time PCR System (Thermo Fisher Scientific) using the HRM MeltDoctor^TM^ reagents (Applied Biosystem). DNA was denatured at 95° for ten minutes, and PCR amplified (45 cycles of 95 °C for 15 s, 60 °C for 60 s and 72 °C for 10 s, and a last high resolution melting step at 95 °C for 15 s, 50 °C for 60 s, 95 °C for 15 s and 60 °C for 30 s) with a combination of a primer forward located in intron 25 (5′-GCAGCTTTTCCACTTATTTTC-3′) and primer reverse located in intron 26 (5′-GGGCGGGACGGGCGCCTTATAATATTCCTTGAGTTTAC-3′). PCR products were directly sequenced with BigDye™ Terminator v1.1 Cycle Sequencing Kit. Sequence products were analysed by capillary electrophoresis (ABI3130XL, Thermo Fisher Scientific).

### 2.5. cDNA Analysis

Total RNA extraction from PBL was performed using PAXgene Blood RNA Kit (Qiagen, Valencia, CA), according to the manufacturer’s instruction. An amount of 500 ng of total RNA were reverse transcribed with Prime-Script RT kit (TaKaRa Biotechnology, Japan) following manufacturer’s protocol. cDNAs were denatured at 94 °C for two minutes, and PCR amplified (33 cycles of 94 °C for 30 s, 58 °C for 30 s, and 72 °C for 45 s, and a final extension step at 72 °C for 10 min) with various combinations of forward primers located in *BRCA2* exon 22 (5′-CTCAGATCCAGTTGGAAAT-3′), exon 24 (5′-GCCCCTTCACTTCAGCAAAT-3′), and exon 25 (5′-AGGGCCACTTTCAAGAGACA-3′), and three reverse primers located in *BRCA2* exon 27: (5′-TCCTTTTGGCCATACAAAGTG-3′FAM), (5′-GCTTTGCAGTTCTTTTGGTCATC-3′) or (5′-TGCAAGTTCTTCGTCAGCT-3′FAM). FAM-labelled PCR products were analysed by capillary electrophoresis (ABI3130, Thermo Fisher Scientific). Size calling was performed with GeneMapper™ Software 5 (Thermo Fisher Scientific) using LIZ-1200 (Thermo Fisher Scientific) as internal size-standard. A detailed description of splicing analysis by RT-PCR and capillary electrophoresis has been described elsewhere (PMID: 24123850). Non-labelled PCR products were directly sequenced (Forward sequence) with BigDye™ Terminator v1.1 Cycle Sequencing Kit. Sequence products were analysed by capillary electrophoresis (ABI3130) with Sequencing Analysis Software v6.0 (Thermo Fisher Scientific).

### 2.6. Diagnostic Algorithm Definition

**Figure 1:**
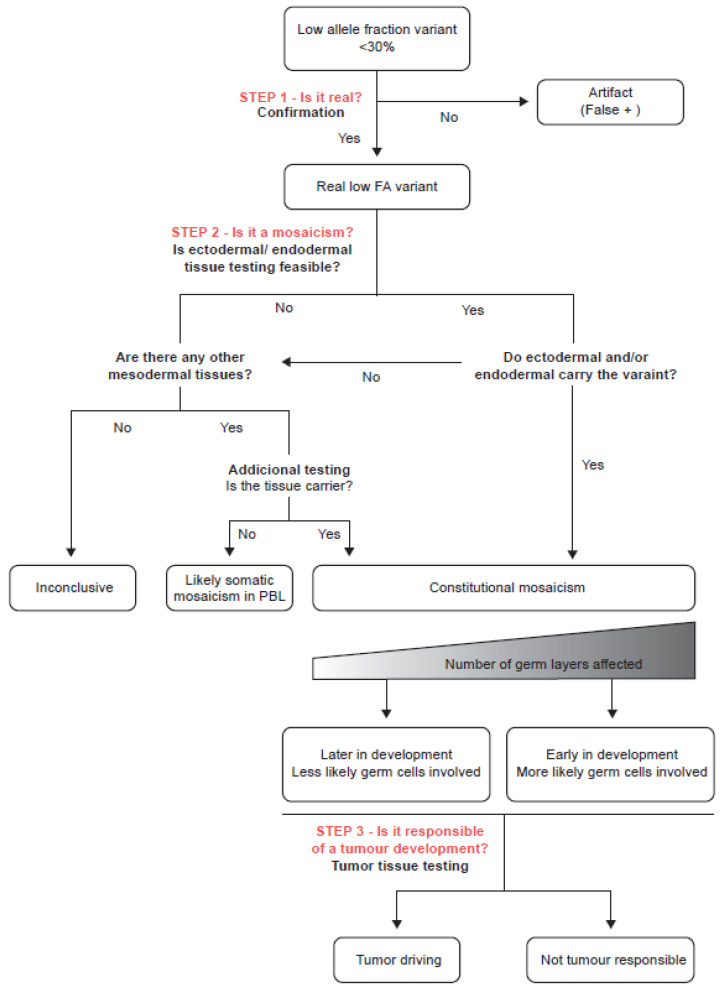
Decisional algorithm tree for low allele fraction variants detected in PBL by NGS. (1) Step 1—Ruling out artefacts by testing the variant with an alternative method. (2) Step 2—Confirming constitutional mosaicism by secondary tissue testing. Since mesodermal tissue has already been tested in blood, secondary tissue testing refers to ectodermal and endodermal tissues testing. Depending on the result, two scenarios are opened: if endodermal or ectodermal tissues are carriers, constitutional mosaicism is confirmed; otherwise, the variant might be confined to blood due to a likely somatic mosaicism in PBL or it can be confined to mesodermal tissues due to a constitutional mosaicism that affects exclusively mesodermal germ layer. To distinguish between both entities, an additional mesodermal tissue testing is required (example ovarian sample). (3) Step 3—Establishing the role of the variant in tumor development by tumor tissue testing.

A diagnostic algorithm for dealing with low VAF was defined. First, the algorithm ensures to rule out false positives results derived from sequencing and alignment artefacts. Follow-up Sanger sequencing or MLPA seems to be the most popular confirmation methods, although variants with VAF below their analytic sensitivity threshold may be missed [17,18]. In these cases, an alternative technique such as digital PCR or deep NGS sequencing may suitable. Second, it allows to discriminate among somatic mosaicism in PBL and constitutional mosaicism. For it, the protocol may include testing and quantifying several tissues derived from additional germ layers. Low invasive samples such as buccal tissue or urine are recommended. Third, it includes tumor testing for the variant to establish its role in tumor development (Figure 1).

## 3. Results

NGS data from peripheral blood DNA was analysed using a customized “HBOC panel” of 18 genes. Twenty-nine variants were called with high-confidence, but none were prioritized according to our prioritization criteria: variants were categorized in the Clinvar database as benign or likely benign by multiple submitters or if they have an MAF > 1%. However, among the low-confidence variants called by the pipeline, a potential candidate *BRCA2* variant stood out. It was the *BRCA2* (NM_000059.3): c.9648+1G>A variant which was marked as low-confidence due to its low variant-fraction (VAF 11%, read depth 625X).

In order to rule out sequencing artefacts, Sanger sequencing was performed that confirmed the presence of the variant in a similar percentage.

As constitutional mosaicism was suspected, three different tissues (buccal swab, urine and breast tumor) were tested for the variant using high depth sequencing (NGS). The analyses revealed the presence of the *BRCA2* c.9648+1G>A variant in 15% of reads in buccal swab, in 23% of the reads in urine and in 66% of reads in breast tumor (Table 1), confirming constitutional mosaicism. NGS sequencing of the paraffin-embedded breast tumor block did not identify additional *BRCA2* mutation as a second hit. Germline *BRCA2* c.9648+1G>A testing in her progenitors was not feasible (adopted proband).

**Figure 2:**
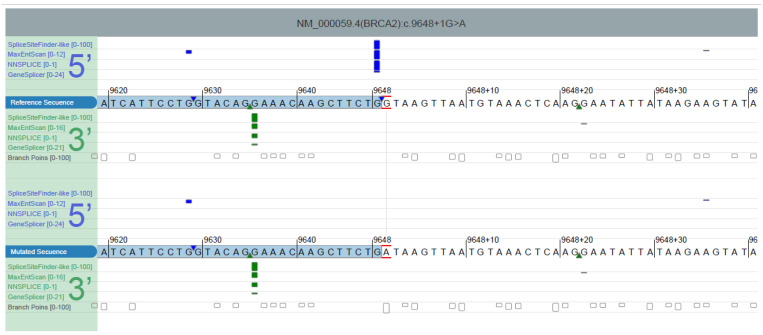
Alamut^TM^ Visual Plus Splicing Predictions. Reference (wild type) and mutated sequences are displayed, and splicing predictions are reported above and under each one. Exons are represented with blue boxes. Hits from SpliceSiteFinder-like, MaxEntScan, NNSPLICE and GeneSplicer are displayed as blue vertical bars for 5′ (donor) sites, and as green vertical bars for 3′ (acceptor) sites. The height of each bar is proportional to the maximum possible score computed by the corresponding algorithm. Known constitutive signals are displayed as small blue (5′) or green (3′) triangles, close to the sequence letters.

This variant consists of a guanidine to adenine substitution in the first nucleotide of intron 26 of the *BRCA2* gene, a position moderately conserved among different species (phyloP score: 5.47 [−19.0,10.9]). It was absent in population databases (GnomAD v2.1.1, 1000 Genomes project v5 and NHLBI Exome Sequencing Project (ESP) v6500) and it was supported to disrupt normal splicing by abolishing a splicing donor site in exon 26. Splicing prediction tools assessed include MaxEntScan [19], Splice Site Prediction by Neural Network (NNSPLICE [20]), Splice Site Finder (SSF-like, based on SSF method by Shapiro and Senapathy, 1987), GeneSplicer [21]) and SpliceAI [22]. They were consulted through Alamut^TM^ Visual Plus (Interactive Biosoftware, Rouen, France) (Figure 2) with the exception of SpliceAI that was consulted online (Table 2).

To gain insight into the pathogenicity of the variant, experimental studies were performed to assess its effect on the transcriptional process of the protein. RNA was obtained from PBL, and reverse transcribed to cDNA by reverse transcription polymerase chain reaction (RT-PCR), allowing the amplification of a cDNA fragment of ~581 nucleotides that encompasses exon 24, 25, 26 and 27 of BRCA2 (gE24Bs-cE27ENIGMA). RNA was obtained from the reported patient along with a healthy control.

**Figure 3:**
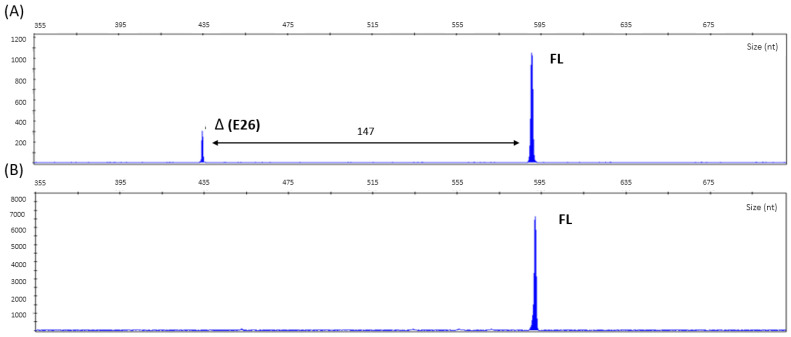
cDNA capillary electrophoresis analysis. Size difference between full-length and alternative splicing transcript is indicated in nucleotides (nt). Panel (**A**) Proband capillary electrophoresis analysis identifies one aberrant transcript. Panel (**B**) Control capillary electrophoresis analysis shows the natural full-length isoform.

RT-PCR products were analysed by capillary electrophoresis, revealing the presence of an aberrant Δ (E26) transcript of ~434 nucleotides in the proband (Figure 3). Size calling of Δ (E26) transcript, 147 nucleotides less than the full-length isoform, is compatible with exon 26 skipping. Additionally, Δ(E26) shows qualitative differences in terms of the relative signal compared with the full-length isoform that is consistent with the mosaic status of the transcript.

**Figure 4:**
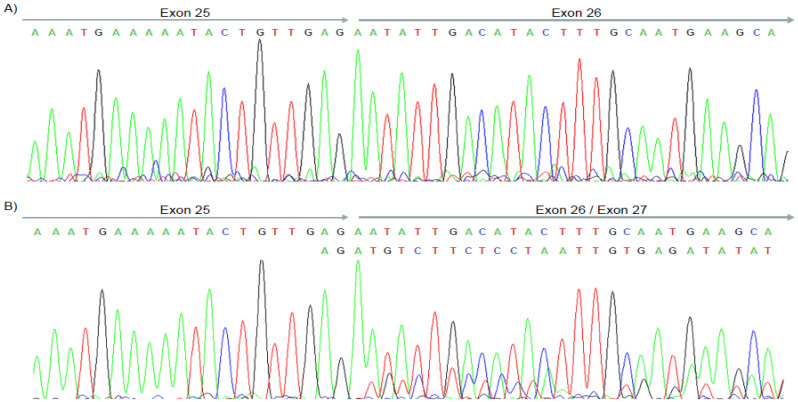
cDNA sequence chromatograms. Panel (**A**) showing Sanger sequencing of a control sample. Sequence displays the junction of exon 25 and 26. Panel (**B**) showing Sanger sequencing of the proband sample. Sequence displays the junction of exon 25 and 26, along with the presence of an overlapping sequence that corresponds to exon 27 sequence.

Suspected exon 26 skipping in the *BRCA2* gene was confirmed by Sanger sequencing of the RT-PCR products. cDNA from the reported patient along with a healthy control were sequenced. Direct sequencing revealed that *BRCA2* c.9648+1G>A spliceogenic variant alters the natural occurring splicing of the junction of exon 25 and 26, leading to the deletion of 147 nucleotides. Deleted sequence belongs to the full sequence of exon 26 and causes an inframe deletion of 46 amino acids (p.Asn3168_Leu3216del) of the *BRCA2* gene (Figure 4).

## 4. Discussion

Somatic mosaicism is a well-known mechanism of cancer caused by post-zygotic events that occur during cell division and affect a specific somatic cell lineage by conferring a growth advantage upon the original clone of a cell [23]. In some cases, the post-zygotic event takes place at an early stage of embryonic development and affects several cell lines. This phenomenon is known as constitutional mosaicism. It can affect tissues derived from any of the three primary germ layers (somatic mosaicism) or it can affect both somatic and germ line cells (gonadosomatic mosaicism). Discriminating between both entities has relevant implications for genetic counselling, given that germinal cells containing the variant can be passed to the offspring.

Constitutional mosaicism has shown to contribute to the aetiology of several cancer predisposition syndromes [24,25,26,27], including HBOC. In 2013, Delon I et al. described for the first time a woman with bilateral basal-like breast cancer caused by a germline mosaic *BRCA1* deletion [8]. Since then, only seven additional cases of constitutional mosaicism in *BRCA1*/2 genes have been reported in HBOC individuals (Table 3), including the one in this study [9,10,11]. The low prevalence of constitutional mosaicism reported in HBOC may be related to low VAF variants being generally overlooked during genetic analysis.

Herein, we report clinical and genetic details of a woman with breast cancer caused by the mosaic c.9648+1G>A variant in *BRCA2*. Genetic diagnostic was reached applying the diagnostic algorithm defined by the laboratory. The c.9648+1G>A variant is absent in the populations’ databases and has already been reported in scientific literature in a woman from a cohort of 7051 Japanese breast cancer patients [28]. Additionally, functional studies conclude that it leads to the skipping of exon 26 of the *BRCA2* protein.

Exon 26 is located in *BRCA2*’s C-terminus DNA-binding domain (DMD), implicated in binding ssDNA and dsDNA. This region is composed of a helical domain and three oligonucleotide/oligosaccharide-binding (OB) folds [29]. The inframe deletion of exon 26 leads to the loss of the last portion of OB3 region, consisting of an α helix of unknown function [30]. A mouse embryonic stem cell (mESC)-based assay [31] revealed that *BRCA2* c.9648+1G>A causes exon 26 skipping (human BRCA2-containing BAC), and that in-frame exon 26 skipping does not complement the removal of the conditional mBrca2 allele, supporting loss-of-function.

This change is classified as pathogenic (Class 5) following the qualitative ACMG [32] criteria since (i) it is absent in several population databases (PM2_Supporting) (ii) it is a GT-AG variant causing an in-frame alteration targeting a region critical to protein function (PVS1_O_Strong) [33] and (iii) a well-stablished functional assay supports damage on gene product (PS3. Additionally, this change is classified as likely pathogenic (Class 4) following the quantitative ACMG point system [34] in which PVS1_strong, PS3 and PM2_Supporting add up to nine points (likely pathogenic: six–nine point range). Consequently, this *BRCA2* variant is most likely responsible for the patient’s clinical phenotype.

The constitutional mosaic nature of the variant was confirmed by assessing samples derived from different germ layers. NGS studies of DNA extracted from PBL (mesodermal origin), urine (endodermal origin) and buccal swab (ectodermal origin) [5] detected the variant in ~10–20% of reads. The consistency of the variation load suggests the variant has most likely arisen de novo as an early postzygotic mutational event, and little variations among tissues suggest that they may had developed from slightly different subpopulations of early embryonic cells and/or that unequal lineage expansions occur later in development [35]. Additionally, NGS sequencing of the paraffin-embedded breast tumor block detected the variant in ~66% of reads, strongly suggesting that this mutation is driving tumor development in this individual.

After reaching the diagnosis, the proband was referred for genetic counselling. Up to date, there are no evidence-based clinical guidelines that allow for accurately quantifying mosaic individual risk and to provide accurate genetic counselling [36]. For this reason, she has been managed as a constitutional heterozygous carrier and the follow-up was established following the SEOM clinical guidelines in hereditary breast and ovarian cancer [37].

Detection of mosaicism constitutes a real diagnostic challenge. On the one hand, methodological limitations may fail to detect low VAF variants, and on the other hand, a lack of habit in low VAF interpretation may result in deleterious variants being discarded and not informed by geneticists. In order to avoid misinterpretations, laboratories should define a diagnostic algorithm for patients presenting with potential mosaic findings identified by NGS.

Quantifying and characterizing constitutional mutations is important not only in establishing a diagnosis, but also in terms of clinical management and in genetic counselling. First, the accurate assessment of several tissues contributes to estimating mosaicism timing that determines the percentage of the affected cells in the organism and the type of tissues involved. Given that carrier tissues may be at risk of developing new tumors, this knowledge would enable for the adjustment of clinical management. Second, an accurate assessment of mutation timing may be useful for guiding genetic counselling. The risk of transmitting a variant is dependent on whether it is present in the germ line and, if so, on the percentage of germ cell progenitor that harbors the mutation [38]. However, currently the low prevalence of constitutional deleterious HBOC-related variants and the unpredictable level of mosaicism within an individual makes specific, management and counselling guideline development difficult.

## 5. Conclusions

To conclude, although mosaicism for *BRCA1* and *BRCA2* variants seems to be rare, low-level mosaic mutations can contribute to the aetiology of breast and ovarian cancer susceptibility. This work proves that c.9648+1G>A carriers are at risk for developing HBOC and highlights the role of mosaic *BRCA1* and *BRCA2* variants in HBOC development. Additionally, this work highlights the need for geneticists to be aware of potential *BRCA1* and *BRCA2* mosaic events during NGS genetic analysis, as failure to detect mosaicism may result in inappropriate genetic counselling and a substantial difference in recurrence risk assessment.

## Figures and Tables

**Table 1:** Tissue distribution of *BRCA2*: c.9648+1G>A variant. Read depth of the position and Variant Allele Fraction (VAF).

Tissue Type	Read Depth	VAF (%)
PBL	625	11
Breast tumour	243	66
Buccal swab	193,704	15
Urine	51,904	23

**Table 2:** SpliceAI scores for splice-site prediction. Scores were computed with the following parameters: genome version GRCh38, score type raw, maximum distance 1000 nucleotides.

∆ Type	∆ Score ^a^	Pre-mRNA Position ^b^
Acceptor Loss	0.70	−147 bp
Donor Loss	1.00	−1 bp
Acceptor Gain	0.00	
Donor Gain	0.01	−21 bp

^a^ Probability that the variant affects splicing at any position within the window around it. ∆ score range from 0 (min) to 1 (max). ^b^ Positions with the biggest change in probability to be splice acceptors or donors. Negative values are upstream (5′) of the variant, and positive are downstream (3′) of the variant.

**Table 3:** *BRCA1* and *BRCA2* constitutionals mosaics.

Case	Age of Diagnosis	Type of Cancer	Nucleotide Change	Amino Acid Change	Gene	VAF	Reference
1	39	Breast	DelEx16		*BRCA1*	~33% PBL and saliva ~36% right breast tumor ~64% left breast tumor	[8]
2	43	Breast	c.1953dupG	p.(Lys652Glufs*21)	*BRCA1*	~5% PBL, buccal swab and non-neoplastic breast ~47% in breast tumor	[2]
3	41	Breast	DelEx1-13		*BRCA1*	~25% PBL	[9]
4	36	Breast	c.9294C>G	p.(Tyr3098Ter)	*BRCA2*	~20 PBL	[3]
						~36% normal breast	
						~21–25% ovary	
						~57% in breast tumor	
5	Mids 50	Ovarian	c.7795G>T	p.(Glu2599Ter)	*BRCA2*	~26% PBL	[10]
						~21–23% buccal swab	
						~77–78% ovarian tumor	
6	42	Ovarian	c.5074G>A	p.(Asp1692Asn)	*BRCA1*	~10% PBL	[11]
						~52% ovarian tumor	
7	53	Ovarian	c.2755_2758dupCCTG	p.(Val920Alafs*6)	*BRCA1*	~14% PBL	[11]
						~59% ovarian tumor	
8	25	Breast	c.9648+1G>A	p.(Asn3168_Leu3216del)	*BRCA2*	~11% PBL	This case
						~15% buccal swab	
						~23% urine	
						~66% breast tumor	

## Data Availability

The datasets generated during and/or analysed during the current study are available from the corresponding author on reasonable request.
